# Paravertebral Blocks in Implant-Based Breast Reconstruction Do Not Induce Increased Postoperative Blood or Drainage Fluid Loss

**DOI:** 10.3390/jcm14061832

**Published:** 2025-03-08

**Authors:** Tonatiuh Flores, Florian J. Jaklin, Martin S. Mayrl, Celina Kerschbaumer, Christina Glisic, Kristina Pfoser, David B. Lumenta, Klaus F. Schrögendorfer, Christoph Hörmann, Konstantin D. Bergmeister

**Affiliations:** 1 Karl Landsteiner University of Health Sciences, Dr-Karl-Dorrek-Straße 30, 3500 Krems, Austriacelina.kerschbaumer@outlook.com (C.K.); christina.glisic@stpoelten.lknoe.at (C.G.); kristina.pfoser@stpoelten.lknoe.at (K.P.); klaus.schroegendorfer@stpoelten.lknoe.at (K.F.S.); christop.hoerman@stpoelten.lknoe.at (C.H.); konstantin.bergmeister@stpoelten.lknoe.at (K.D.B.); 2 Clinical Department of Plastic, Aesthetic and Reconstructive Surgery, University Clinic of St. Poelten, 3100 St. Poelten, Austria; 3 Clinical Laboratory for Bionic Extremity Reconstruction, University Clinic for Plastic, Reconstructive and Aesthetic Surgery, Medical University of Vienna, 1090 Vienna, Austria; florian.jaklin@meduniwien.ac.at; 4 Division of Plastic, Aesthetic and Reconstructive Surgery, Department of Surgery, Medical University of Graz, 8010 Graz, Austria; 5 Clinical Department of Anesthesiology and Intensive Care Medicine, University Clinic of St. Poelten, 3100 St. Poelten, Austria

**Keywords:** breast cancer, breast reconstruction, pain catheter, postoperative blood loss, drainage volume, patient after-care

## Abstract

**Background**: Women undergoing a mastectomy often suffer severely from the sequelae of losing one or both breasts. Implant-based breast reconstruction restores female body integrity but can result in significant postoperative pain. The use of paravertebral catheters has been shown to aid significantly in pain management during the postoperative recovery. However, the vasodilation that is induced by paravertebral blocks may lead to prolonged drainage fluid secretion, blood loss and increased likelihood of revision surgery. Therefore, we analyzed the effects of paravertebral blocks after combined mastectomy and immediate breast reconstruction. **Methods**: We analyzed 115 breast surgeries at the department of Plastic Surgery at the University clinic of St. Poelten between 1 August 2018 and 31 December 2022. Patients were analyzed regarding postoperative hemoglobin loss and drainage fluid volumes and their correlation with paravertebral blocks. Statistical analyses were performed using Levene’s Test for Equality of Variances within our cohort. **Results**: The postoperative hemoglobin loss did not differ significantly between our groups (*p* = 0.295). Furthermore, a paravertebral block did not increase the amount of postoperative drainage fluid volumes (*p* = 0.508). Women receiving paravertebral blocks also did not stay longer in hospitals (*p* = 0.276). No paravertebral block-associated complication was seen. **Conclusions**: In this study, we demonstrated paravertebral blocks to be safe adjuncts in breast reconstruction to minimize pain without leading to increased blood loss or seroma formation. This indicated that vasodilatation induced by paravertebral blocks did not negatively influence the postoperative recovery. In conclusion, postoperative pain management using paravertebral blocks can be a beneficial therapeutic adjunct in surgical management of breast cancer patients.

## 1. Introduction

Mastectomy due to breast cancer severely impacts the well-being of affected women on account of a loss of body integrity [1,2,3,4,5]. Consequently, breast reconstruction represents an essential pillar of modern breast cancer treatment to reduce the suffering of breast cancer patients [6,7,8,9]. During surgery, the primary goal is complete tumor resection, and thus, the residual skin often experiences severe perfusion disturbances, which can negatively affect the direct implant insertion [10,11,12,13,14,15]. Here, submuscular expanders help to generate sufficient soft tissue for further reconstruction without stressing the residual skin [16,17,18]. However, their placement entails painful muscle stretching, leading to postoperative discomfort and pain [19,20,21,22].

Since the introduction of paravertebral catheters, postoperative pain has decreased noticeably for women after a mastectomy [23,24,25,26,27]. Its popularity has increased over the past decades and is now part of the advanced perioperative armamentarium after mastectomy [26,28,29]. Yet, paravertebral blocks (PVBs) are associated with several undesired issues [30,31,32,33,34]. Pneumothorax, as its most commonly encountered complication, dislocation and occlusion are frequently seen [30,31,32,34]. And while the numbing of pain receptors is the primary goal, vasodilation is simultaneously provoked due to the sympathicolysis [35]. Whilst vasodilatation is a desired effect in replantation surgery, it may result in prolonged bleeding or an increased postoperative drainage fluid volume and thus lead to complications [36,37,38,39].

In this paper, we analyzed the effect of paravertebral blocks on the postoperative blood loss and drainage fluid volume in women undergoing breast reconstruction after mastectomy. Our aim was to investigate the impact of vasodilation on the postoperative hemoglobin levels and drainage fluid volumes. To our knowledge, this is the first study investigating the effect of paravertebral catheters on breast cancer patients after mastectomy.

## 2. Materials and Methods

### 2.1. Study Design and Patient Analysis

In this study, we analyzed patients undergoing subcutaneous mastectomy and consecutive breast reconstruction at the Clinical Department for Plastic, Aesthetic and Reconstructive Surgery at the University Hospital St. Poelten between 1 August 2018 and 31 December 2022. This study was conducted as a retrospective single-center study. Ethical approval was obtained from the local institutional review board at the Karl Landsteiner University of Health Sciences Krems (reference number: ECS 1085/2023). Analyzed factors included the patients’ age at surgery, BMI, mastectomy weight, mastectomy side (unilateral, bilateral), sentinel lymph node dissection, axillary dissection, perioperative hemoglobin and hematocrit, postoperative drainage fluid volume, operation time and duration of hospital stay.

Hemoglobin values (g/dL) were analyzed prior to surgery and on the first postoperative day. Anemia was defined as values below 12 g/dL according to the WHO classification [39]. The drainage output was documented every 12 h until the removal of the drainage catheters. Drainage removal was conducted if the output was less than 30 mL in 24 h. The patients included were divided into two groups, based on whether they were receiving paravertebral block (paravertebral block (PVB) group) or not (non-paravertebral block (non-PVB) group). Every patient at our department was offered PVB. Women accepting and then receiving PVB were added to the PVB group. In case of PVB rejection by patients, PVB was not installed. Thus, patients were transferred to the non-PVB group.

None of the included patients displayed any kind of liver abnormalities, hematopoietic disorders, or diseases in need of immunomodulatory medication. Further, neoadjuvant chemo-, radiation- and hormone therapies were analyzed in terms of their influence on blood loss or drainage fluid volume in this study.

### 2.2. Paravertebral Block

**Figure 1:**
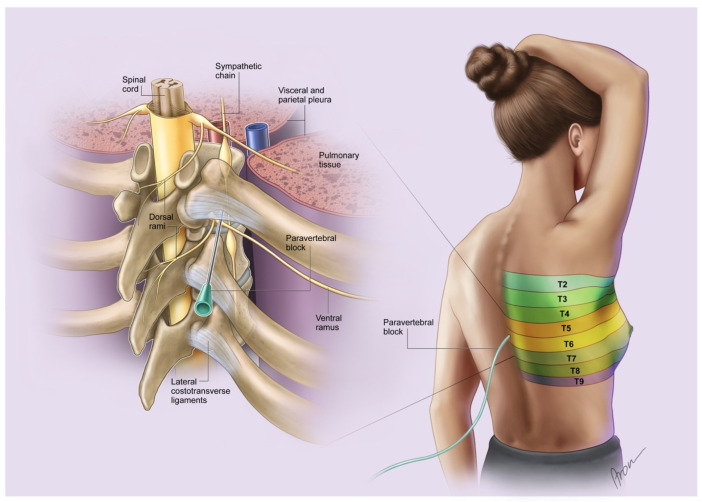
PVB in place blocking the respective dermatomes for sufficient pain relief after mastectomy. Note the proximity of the catheter to the sympathetic chain. The segments T2–T9 indicate sensory innervated dermatomes.

Every patient at our department who was scheduled for mastectomy was offered a paravertebral block for adequate and facilitated postoperative pain management. The procedure was ultrasound-guided and performed under sterile conditions on the day before surgery by an anesthesiologist who is specifically trained for this intervention. The installation of the paravertebral catheters was conducted either with the patient sitting or in a lateral decubitus position at the level of Th 4. After successful installation, continuity testing was performed with two milliliters of Ropivacain (Ropinaest^®^, Gebro Pharma, Bahnhofbichl 11, 6391 Fieberbrunn, Germany) (Figure 1). The PVB cable was fixed with transparent occlusion foil to be able to fully review the catheter at least once a day and to prevent unintentional dislocation.

Additionally, a chest X-ray was performed for pneumothorax exclusion. Each catheter was connected to an ON-Q^®^ pump (On-Q^®^ pain relief system, AVANOS Medical, c/o Pier 11, Schauenburgerstraße 10, 20095 Hamburg, Germany) with a select-a-flow variable rate controller with a reservoir of 400 mL, containing Ropivacain. The dosage settings were at 2, 4, 6, 8, 10, 12 or 14 mL/h, individually adjusted to the patient’s pain level. Each paravertebral catheter was injected with two milliliters of Ropivacain (Ropinaest^®^, Gebro Pharma 6391 Fieberbrunn, Germany) 30 min before the incision.

Catheters were reduced starting on the second postoperative day by 2 mL/h each day until reaching 0 mL/h. The standard postoperative dosage for postoperative catheter influx was 10 mL/h. PVBs were checked by anesthesiologists daily until removal.

### 2.3. Operative Procedure

Subcutaneous mastectomies were conducted either through lateral incision or in case of a simultaneous reduction via inverted T incision. A retromammillary cylinder was additionally retrieved and sent for frozen section examination to determine whether the NAC (nipple areolar complex) had to be removed or not. If the sentinel lymph node tested positive intraoperatively, axillary dissection was performed after the mastectomy. If the retromammillary cylinder tested positive intraoperatively, the NAC was removed.

In case of sufficient subcutaneous tissue, immediate prepectoral breast reconstruction, using Mentor implants (MENTOR^®^ Contour Profile Gel™ (CPG™), Mentor Worldwide LLC, 31 Technology Drive, Suite 200, Irvine, CA 92618, USA) with a textured surface and Serasynth^®^ Mesh (Serag-Wiessner GmbH & Co. Kg Zum Kugelfang 8–12, 95119 Naila, Germany), was performed.

If the subcutaneous layers did not seem resilient enough for prepectoral implant placing, submuscular tissue expanders were installed. All implants had textured and anatomical properties (Mentor Siltex^®^ Contour ProfileTM BeckerTM 35 Expander, Mentor Worldwide LLC, 31 Technology Drive, Suite 200, Irvine, CA 92618, USA). Submuscular pocket preparation was performed through incising the major pectoral muscle parallel to its muscle fiber course, approximately at the level of the fourth to fifth intercostal space. The serratus anterior fascia was partially raised to support the implant inferiorly and laterally if needed. Port systems were installed at the level of the anterior axillary line at the level of Th 5. If no axillary dissection was performed, subcutaneous and submuscular drains were placed. In case of axillary dissection, one additional drain was inserted in the axillary wound cavity. Drains were removed in case of less than 30 mL of fluid within 24 h.

### 2.4. Statistics and Data Management

The endpoint of our analyses was to assess the hemoglobin loss and the volume of the postoperative drainage fluid after mastectomy. Our dataset was divided into two groups: women with and women without paravertebral block. All data were reported anonymously. The data protection management complied with Austrian legislation. Data collection and processing were performed with Microsoft Excel (Microsoft corp., Washington, DC, USA), and statistical analyses were performed using IBM SPSS Statistics version 29 (©IBM, Armonk, NY, USA). Nominal data are described using absolute frequencies and percentages. For metric data, the mean and standard deviation are indicated. To correlate the amount of postoperative drainage fluid volume and hemoglobin loss to paravertebral blocks, correlation analyses using independent samples Mann–Whitney U Tests were performed. Further, paired *t*-test analyses were conducted to compare groups, specifically regarding the postoperative drainage volumes of patients with and without paravertebral block. A two-sided *p* ≤ 0.05 was regarded as statistically significant.

## 3. Results

In total, 1128 breast surgeries were analyzed within this study. Of these, 432 were excluded due to being body forming surgeries, 119 due to being breast implant revisions, 142 due to being second-stage reconstruction, 215 due to being non-implant-based cancer-related breast surgeries, and 3 due to being sole tissue expander implantations. Additionally, 65 surgeries had to be excluded due to a lack of sufficient data. Finally, 152 mastectomies in 115 patients with consecutive breast reconstruction met our criteria and were included in this study.

Here, 124 tissue expanders and 28 definitive breast implants were implanted in 115 patients. In our study group, 52 (45.22%) women received preoperative paravertebral catheter, and 63 (54.78%) underwent surgery without paravertebral block (Table 1).

### 3.1. Patient Demographics

The mean overall patient age at surgery was 47.50 years ± 10.82 years, ranging from 23 to 76 years (Table 1). The mean BMI was 28.84 kg/m^2^ ± 4.59 kg/m^2^, ranging from 16.4 to 41.1 kg/m^2^. The mean duration of surgery was 175.82 min ± 48.79 min, varying from 85 to 329 min. The mean hospital stay was 8.71 days ± 2.21 days, ranging from 4 to 18 days.

The mean total mastectomy weight was 636.32 ± 415.48 g, ranging from 285 to 2543 g. The mean unilateral mastectomy weight on the left side was 319.59 ± 329.30 g, with a minimum weight of 84 g and a maximum weight of 1805 g. The mean unilateral mastectomy weight on the right side was 316.73 ± 318.12 g, ranging from 70 to 1286 g in total. Women without PVB experienced bilateral mastectomy in 24 (27.59%) cases and women with PVB in 13 (20.97%) cases. Overall, 78 (67.83%) patients underwent sentinel lymph node dissection. In addition, 64 (82.05%) were unilateral, and 7 (17.95%) were bilateral. A total of 36 (31.30%) patients received axillary dissection. Of these, 16 (44.44%) were left-sided, 18 (50%) were right-sided, and 1 (5.56%) was bilateral.

The mean overall Hb loss was 2.55 ± 1.54 g/dL (Table 1). Preoperative anemia was seen in 28 (33.05%) of our patients. The mean preoperative Hb levels were 13.04 g/dL ± 1.37 g/dL. Postoperatively, 100 (86.96%) patients showed hemoglobin levels below 12 g/dL. The mean postoperative Hb levels were 10.49 ±1.71 g/dL overall.

#### 3.1.1. Non-PVB Group

**Figure 2:**
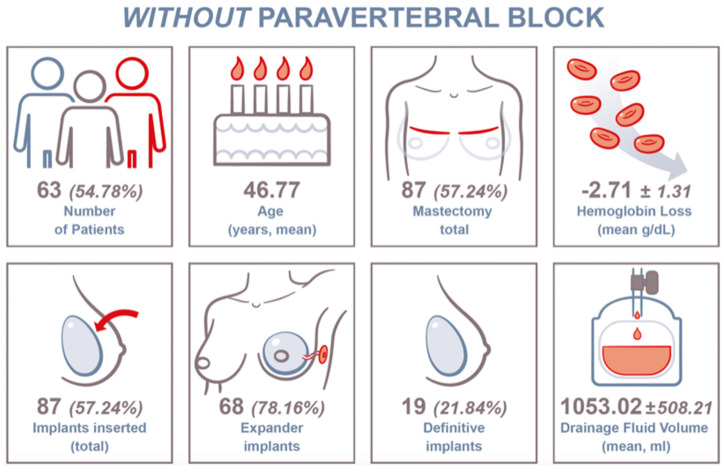
Key data chart for included patients without paravertebral block (PVB) and clinical findings.

In total, 63 (54.78%) patients were included in this group. The mean patient age at surgery was 46.77 years ± 11.08 years. In this group, 87 (57.24%) mastectomies were performed. Consecutively, 68 (78.16%) tissue expanders and 19 (21.84%) definitive breast implants were installed. The mean Hb loss was 2.71 ± 1.31 g/dL. The mean drainage fluid volume was 1053.02 ± 508.21 mL (Figure 2).

In total, 23 (36.51%) women received neoadjuvant chemotherapy, 7 (11.11%) underwent neoadjuvant radiation therapy, and none had neoadjuvant hormone therapy.

#### 3.1.2. PVB Group

**Figure 3:**
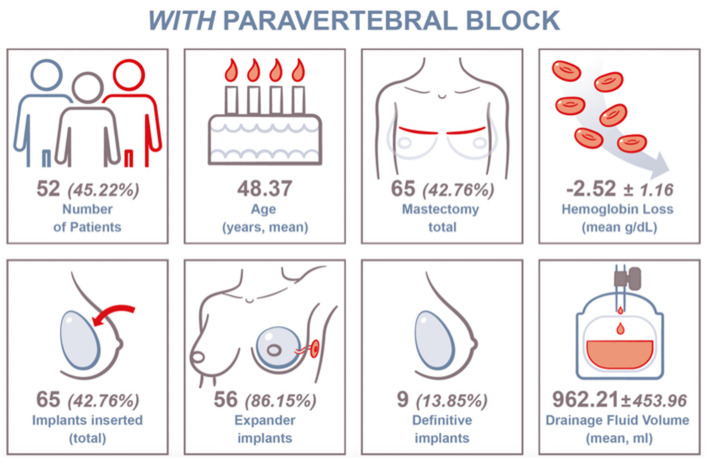
Key data chart for included PVB patients and clinical findings.

In total, 52 (45.22%) patients were included in this group. The mean patient age at surgery was 48.37 years ± 10.54 years. In this group, 65 (42.76%) mastectomies were performed. Consecutively, 56 (86.15%) tissue expanders and 9 (13.85%) definitive breast implants were installed. The mean Hb loss was 2.52 ± 1.16 g/dL. The mean drainage fluid volume was 962.21 ± 453.96 mL (Figure 3).

In total, 21 (40.38%) women received neoadjuvant chemotherapy, 4 (7.7%) underwent neoadjuvant radiation therapy, and none had neoadjuvant hormone therapy.

### 3.2. Hemoglobin Levels

**Figure 4:**
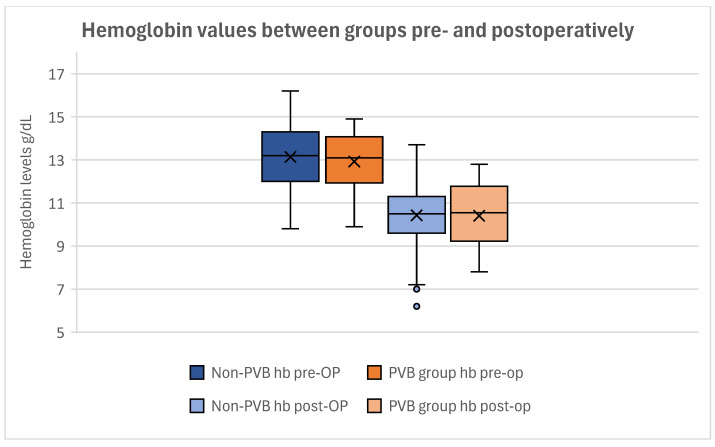
Boxplot of the hemoglobin levels of the groups. The preoperative values can be seen on the left in darker colors (dark blue: non-PVB group; dark orange: PVB group). The postoperative hemoglobin levels can be seen on the right in brighter colors (light blue: non-PVB group; light orange: PVB group). Although a clear difference in pre- and postoperative values is shown, no statistical significance could be proven. Outliers are depicted as dots.

The hemoglobin levels did not differ significantly between the groups. The mean preoperative Hb level in women without PVB was 13.14 ± 1.40 g/dL. Women with PVB showed a mean preoperative Hb level of 12.92 ± 1.30 g/dL (Figure 4). Postoperatively, no significant difference was seen either, as the mean postoperative Hb was 10.42 ± 1.41 g/dL in the non-PVB group and 10.41 ± 1.38 g/dL in the PVB group (Figure 4).

Conducting a *T*-Test for the Equality of Means, no significant difference was found between the preoperative and postoperative hemoglobin levels between our groups (p_preoperative_ = 0.404; p_postoperative_ = 0.615) (Table 2).

**Figure 5:**
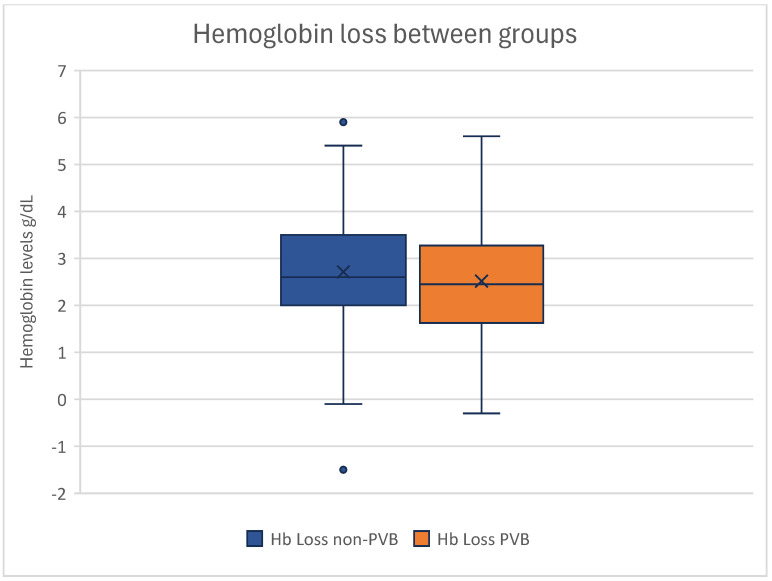
Boxplot of hemoglobin losses of groups. Non-paravertebral block patients can be seen on the left, and patients with paravertebral block are depicted on the right. No statistical significance can be seen in the difference in hemoglobin drop between the groups. Outliers are displayed as dots.

The mean Hb loss was 2.71 ± 1.31 g/dL in the non-PVB group and 2.52 ± 1.16 g/dL in the PVB group (Figure 5).

The *T*-Test for Equality of Means showed no statistical significance in Hb loss between our groups (*p* = 0.295) (Table 3).

Correlating the influence of PVB on the postoperative Hb loss, no statistical difference could be observed (*p* = 0.397), demonstrating that paravertebral catheters had no significant impact on the perioperative hemoglobin loss within our cohort.

### 3.3. Drainage Fluid Volume

**Figure 6:**
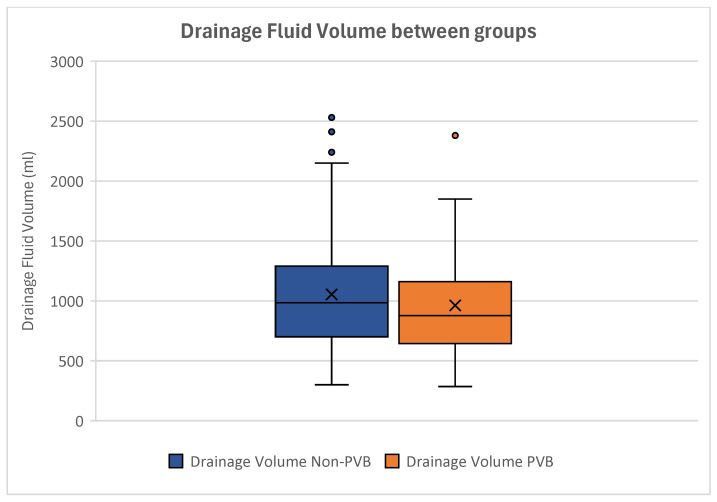
Boxplot showing the drainage fluid volumes of the groups. Non-paravertebral block patients can be seen on the left, while patients with paravertebral block are depicted on the right. No significant difference can be observed between the groups regarding postoperative drainage fluid loss. Outliers are displayed as dots.

The mean drainage fluid volume was 1053.02 ± 508.21 mL in patients without paravertebral block and 962.21 ± 453.96 mL in women with paravertebral block (Figure 6).

The *T*-Test for Equality of Means displayed no statistical difference between the postoperative drainage fluid volumes (*p* = 0.508) (Table 4).

Conducting independent samples analyses, no significant influence of PVB on the postoperative drainage fluid volume could be seen (*p* = 0.367), demonstrating that the drainage fluid volume was not affected by paravertebral catheters.

### 3.4. Duration of Surgery

**Figure 7:**
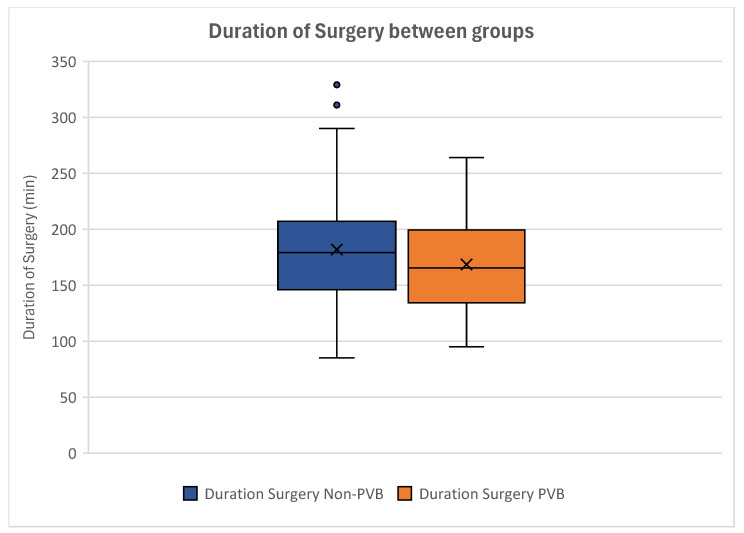
Boxplot of the durations of surgery of the groups. Women without paravertebral block experienced longer operation times. Non-paravertebral block patients can be seen on the left (blue), while patients with paravertebral block are depicted on the right (orange). Outliers are displayed as dots.

Women without paravertebral block showed a mean surgery time of 181.81 ± 53.02 min. The mean duration of surgery was 168.56 ± 41.97 min in patients with PVB (Figure 7).

Our analyses displayed no significant difference regarding the duration of surgery between our groups. By conducting a *T*-Test for Equality of Means, this was statistically proven (*p* = 0.150) (Table 5).

Analyzing the correlation of paravertebral blocks and the duration of surgery, no statistical significance could be seen (*p* = 0.260). This indicated that women receiving paravertebral blocks did not experience a longer duration of surgery than women without paravertebral block.

### 3.5. Hospital Stay

**Figure 8:**
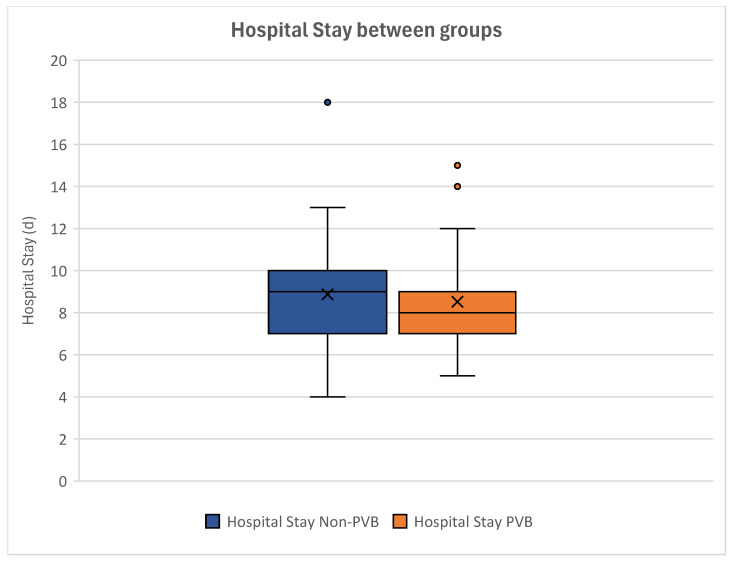
Boxplot of the lengths of hospital stay of the groups. Non-paravertebral block patients can be seen on the left, while patients with paravertebral block are depicted on the right. The duration of in-hospital stay did not differ significantly between groups. Outliers can be seen as dots.

Women without paravertebral catheters showed a mean in-hospital duration of 8.87 days ± 2.30, whereas women with paravertebral block stayed in hospital for a mean of 8.52 days ± 2.07 (Figure 8).

Our analyses showed no statistical significance in the duration of hospital stay between women with and without paravertebral catheters (*p* = 0.348) (Table 6).

Further, no statistical significance between our groups could be seen after performing correlation analyses (*p* = 0.275). This showed that paravertebral block had no influence on the duration of hospital stay.

### 3.6. Neoadjuvant Breast Cancer-Related Therapy

Women without PVB had neoadjuvant chemotherapy in 23 (36.51%) cases, whereas patients receiving PVB underwent neoadjuvant chemotherapy in 21 cases (40.38%).

Neoadjuvant radiation therapy was performed in 7 (11.11%) cases in the non-PVB group and in 4 (7.7%) case in the PVB group. Performing independent *t*-testing, no statistical significance could be observed, neither in neoadjuvant chemotherapy (*p* = 0.674) or radiation therapy (*p* = 0.229).

Conducting correlation analyses, we did not see any correlation of neoadjuvant chemo- or radiation therapy with blood loss (p_chemo_ = 0.977; p_radio_ = 0.504). Correlating the drainage volume to radiation therapy, similarly, showed no statistical significance (*p* = 0.800). When analyzing chemotherapy, however, a significant correlation could be observed with the drainage fluid volume (*p* = 0.004).

## 4. Discussion

Breast cancer is the most common malignancy in women [40,41,42,43]. Although modern diagnostics enable early detection, it often requires mastectomy [44,45,46,47]. Thus, breast reconstruction is often needed to prevent the negative after-effects of breast loss [1,48,49]. To facilitate postoperative recovery, paravertebral blocks are used frequently in terms of postoperative pain management [26,29,30,50,51]. This enables early mobilization and shorter hospital stays [33,52,53]. The complications of PVBs are well known and include pneumothorax, pain at the puncture site and dislocation but are generally rare [36,37,38,54]. However, little is known of their effects on postoperative recovery and blood loss [28,33,52,53].

**Figure 9:**
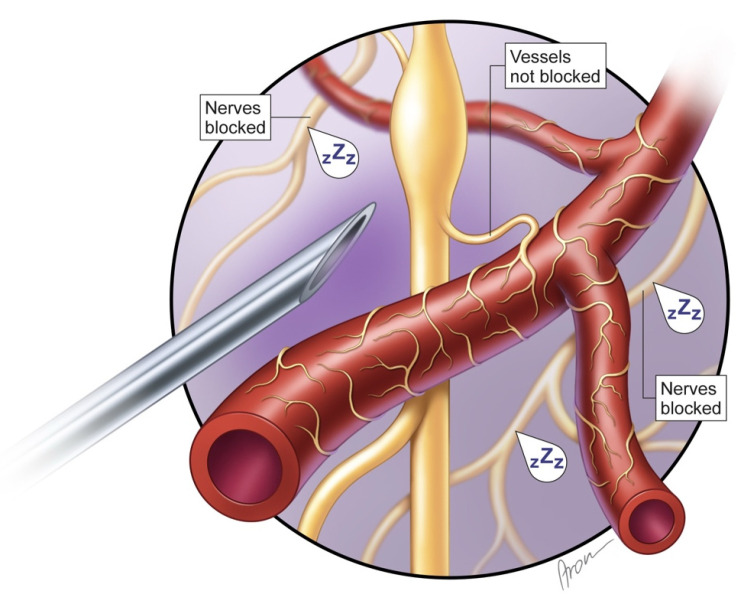
PVB block demonstrating that although pain fibers (marked as “zZz”) are blocked, vessel innervation remains unhindered (vessels are not blocked).

Nerval blocks are frequently used in replantation surgery, as their vasodilatory effect has proven to be beneficial for limb perfusion [35,36,37,38,39]. This clinical effect suggests that neural blockade interrupts the innervation of vessels, causing vasodilation and thus increasing, e.g., limb perfusion and supporting replantation success. Consequently, it is suspected that paravertebral blocks may also induce vessel dilation and therefore might increase the drainage fluid loss or blood loss. It is assumed that the blockade of sympathetic fibers on vessels sustaining breast perfusion also results in vasodilatory effects, which is made use of in case of limb replantation. Nonetheless, this was not seen in our results. The mean hemoglobin loss did not differ significantly (*p* = 0.295) between groups; moreover, the postoperative hemoglobin levels where similar among our cohort (p_postoperative_ = 0.604). Thus, the application of PVB neither led to relevant bleeding at the point of insertion nor at the mastectomy site. Therefore, we can presume that PVB only inhibit nerve fibers that are responsible for pain conduction, which has already been proven by several studies [23,24,25,26,27] (Figure 9).

The drainage fluid volume was additionally supposed to increase with elevated blood perfusion, as more blood circulation further entails a fluid shift into the extracellular matrix. Nonetheless, this could also not be observed in our study. Here, the postoperative drainage fluid volumes showed no significant difference (*p* = 0.508) and therefore did not lead to delayed drainage removal or prolonged in-hospital treatment.

Overall, women with PVB did not experience any disadvantage in perioperative recovery, as no complications were encountered in relation to paravertebral blocks. Although not quantified, we observed that our patients experienced less pain and tolerated dressing changes better with PVBs installed. These effects of paravertebral blocks are already well known and significantly support breast cancer patients while recovering from surgery [24,26,29,51,55,56]. Consequently, we have implemented the use of paravertebral blocks in our preoperative schedules when planning mastectomies.

However, a few challenges were encountered with PVBs in the daily routine, arising from organizational matters. PVBs require adequately trained anesthesiologists, the coordination of their installation in a specialized setting (ultrasound-guided, trained staff, etc.) and time (approximately 30–60 mins). Also, daily follow-up of proper functionality is required, and early mobilization may sometimes lead to catheter disconnection. Further, a more fundamental insight into the relationship between PVBs and postoperative opioid usage should be achieved in the future.

Additionally, breast cancer staging, as well as hormonal cancer signatures, ought to be addressed in further studies. And although included in our analyses, the relationship between neoadjuvant chemo- and radiation therapy and blood loss and drainage fluid volume is an interesting field of research. Besides chemotherapy appearing to significantly correlate with the drainage fluid volume, unfortunately, this correlation holds diminished value due to its small sample size.

Nevertheless, we consider the usage of paravertebral blocks in post-mastectomy recovery beneficial for our patients. They have been shown to significantly ameliorate patients’ postoperative recovery and to reduce the burden put on patients by cancer treatment.

## 5. Conclusions

Women receiving paravertebral blocks did not experience any disadvantages regarding surgical procedure or postoperative recovery, while early mobilization was possible. Substantiated by our findings, we recommend including paravertebral blocks in the basic armamentarium when performing mastectomies, as their conduction is safe and feasible.

## Figures and Tables

**Table 1:** Demography of patients included in this study.

Patient Characteristics		Without PVB	With PVB	Overall Patients	*p*-Values
Number		63 (54.78%)	52 (45.22%)	115	
	Mean	46.77	48.37	47.5	
Age (years)	Min–Max	23–76	29–70	23–76	0.438
	STD	±11.08	±10.54	±10.82	
	Mean	25.40	24.16	24.84	
BMI (kg/m^2^)	Min–Max	16.4–41.1	17.9–37.5	16.4–41.1	0.152
	STD	±4.58	±4.46	±4.59	
	Mean	181.81	168.56	175.82	
Duration of surgery (min)	Min–Max	85–329	95–264	85–329	0.150
	STD	±53.02	±41.97	48.79	
	Total	87 (57.24%)	65 (42.76%)	152	
Number of inserted implants	Expanders	68 (78.16%)	56 (86.15%)	124 (81.49%)	0.133
	Def. Implants	19 (21.84%)	9 (13.85%)	28 (18.41%)	
	Mean	8.87	8.52	8.71	
Hospital stay (days)	Min–Max	4–18	5–15	4–18	0.396
	STD	±2.30	±2.07	±2.21	
Mastectomies total		87 (57.24%)	65 (42.76%)	152	0.204
Sentinel lymph node	Total	39 (50%)	39 (50%)	78	0.234
Axillary dissection	Total	24 (66.67%)	12 (33.33%)	36	0.104
	Mean	13.14	12.92	13.04	
Hb pre-op (g/dL)	Min–Max	9.8–16.2	9.9–14.9	9.8–16.2	0.404
	STD	±1.40	±1.30	±1.37	
	Mean	10.42	10.41	10.49	
Hb post-op (g/dL)	Min–Max	7–10.3	10.2–11.8	7–11.8	0.615
	STD	±1.41	±1.38	±1.71	
	Mean	−2.71	−2.52	−2.55	
Hb Difference pre-post	Min–Max	−5.9–+7.1	−5.6–+0.3	+0.3–−5.9	0.856
	STD	±1.31	±1.16	±1.54	
	Mean	1053.02	962.21	997	
Drainage volume (mL)	Min–Max	300–2530	285–2380	285–2530	0.323
	STD	±508.21	±453.96	±496.39	
	Mean	7.38	7.25	7.32	
Drainage inlay time (d)	Min–Max	3–11	4–14	3–14	0.957
	STD	±1.91	±1.95	±1.93	

**Table 2:** *T*-Test for Equality of Means regarding hemoglobin difference between our groups, preoperatively and postoperatively. Our analyses showed no significant difference between the pre- and postoperative hemoglobin levels of the groups (p_preoperative_ = 0.404; p_postoperative_ = 0.615).

*T*-Test for Equality of Means							
	F	Sig.	T	df	One-Sided *p*	Two-Sided *p*	Mean Difference
Hemoglobin preoperative	0.087	0.769	0.838	113	0.202	**0.404**	0.2151
Hemoglobin postoperative	0.026	0.873	0.504	113	0.308	**0.615**	0.1624
			0.520	110.990	302	0.604	0.1624

**Table 3:** *T*-Test for Equality of Means showing no significance in the difference between pre- and postoperative hemoglobin values (*p* = 0.295). This shows that sympathicolysis makes no significant contribution to the postoperative hemoglobin loss.

*T*-Test for Equality of Means							
	F	Sig.	T	df	One-Sided *p*	Two-Sided *p*	Mean Difference
Hemoglobin Loss	0.135	0.714	1.045	115	0.149	**0.295**	0.243

**Table 4:** *T*-testing of independent samples showing no significant difference in postoperative drainage fluid volumes between women with and without paravertebral block (*p* = 0.508). This demonstrates that postoperative drainage fluid volumes are not affected by paravertebral catheters and consecutive sympathicolysis.

*T*-Test for Equality of Means							
	F	Sig.	T	df	One-Sided *p*	Two-Sided *p*	Mean Difference
Drainage fluid volume	0.388	0.543	0.665	115	0.254	**0.508**	61.788

**Table 5:** *T*-Test for Equality of Means demonstrating no statistical significance in the duration of surgery between the groups (*p* = 0.150).

*T*-Test for Equality of Means							
	F	Sig.	T	df	One-Sided *p*	Two-Sided *p*	Mean Difference
Duration of Surgery	0.682	0.411	1.450	113	0.075	**0.150**	13.251

**Table 6:** Independent sample *T*-testing of lengths of hospital stay of both groups. Here, no statistical significance between the in-hospital duration can be observed (*p* = 0.348). This shows that women with or without PVB were admitted for a similar time-period.

*T*-Test for Equality of Means							
	F	Sig.	T	df	One-Sided *p*	Two-Sided *p*	Mean Difference
Hospital stay	0.823	0.366	0.947	115	0.173	**0.348**	0.388

## Data Availability

All the data analyzed during the current study are available from the corresponding author on reasonable request.
